# THE PROTEOMICS OF LONGEVITY

**DOI:** 10.1093/geroni/igz038.760

**Published:** 2019-11-08

**Authors:** Eric Orwoll, Jack Wiedrick, Steven R Cummings, Dan Evans, Wanlin Zheng, Cory Funk, Nathan Price, Jodi Lapidus

**Affiliations:** 1 Oregon Health & Sciences University, Oregon, United States; 2 OHSU, Portland, Oregon, United States; 3 San Francisco Coordinating Center, San Francisco, California, United States; 4 Institute for Systems Biology, Seattle, Washington, United States

## Abstract

The biological underpinnings of longevity are poorly elucidated in humans. We used a novel, high-throughput discovery-proteomics approach to identify serum proteins associated with longevity (living beyond 90th percentile of survival) in community-dwelling men age ≥65 years in the MrOs Study. Baseline serum from 2473 men was analyzed using liquid chromatography-ion mobility-mass spectrometry. >21,000 peptides and 2931 proteins were recognized. Twenty-five proteins significantly associated with attainment of longevity over 15 yrs of observation were identified using rigorous statistical methods. 25 proteins were significantly associated; all were lower in long lived men than in men dying earlier. Most longevity-associated proteins were from inflammatory pathways; some have multifunctional biological roles potentially reflecting other mechanisms. Pathway analyses suggest important upstream regulators may be causally responsible for the associations. These results provide the opportunity to evaluate these proteins as biomarkers, and highlight the potential importance of their biological pathways in the origins of long life.

